# Epigenetic regulation of retinal development

**DOI:** 10.1186/s13072-021-00384-w

**Published:** 2021-02-09

**Authors:** Reza Raeisossadati, Merari F. R. Ferrari, Alexandre Hiroaki Kihara, Issam AlDiri, Jeffrey M. Gross

**Affiliations:** 1 Departamento de Genética E Biologia Evolutiva, Instituto de Biociencias, Universidade de Sao Paulo, Rua Do Matao, 277, Cidade Universitaria, Sao Paulo, SP 05508-090 Brazil; 2 Centro de Matemática, Computação E Cognição, Universidade Federal Do ABC, Santo André, Brazil; 3 Departments of Ophthalmology and Developmental Biology, Louis J. Fox Center for Vision Restoration, University of Pittsburgh School of Medicine, Pittsburgh, PA USA

**Keywords:** Epigenetics, Retina, Histone, DNA methylation, lncRNA, Development, Chromatin

## Abstract

In the developing vertebrate retina, retinal progenitor cells (RPCs) proliferate and give rise to terminally differentiated neurons with exquisite spatio-temporal precision. Lineage commitment, fate determination and terminal differentiation are controlled by intricate crosstalk between the genome and epigenome. Indeed, epigenetic regulation plays pivotal roles in numerous cell fate specification and differentiation events in the retina. Moreover, aberrant chromatin structure can contribute to developmental disorders and retinal pathologies. In this review, we highlight recent advances in our understanding of epigenetic regulation in the retina. We also provide insight into several aspects of epigenetic-related regulation that should be investigated in future studies of retinal development and disease. Importantly, focusing on these mechanisms could contribute to the development of novel treatment strategies targeting a variety of retinal disorders.

## Introduction

**Fig. 1:**
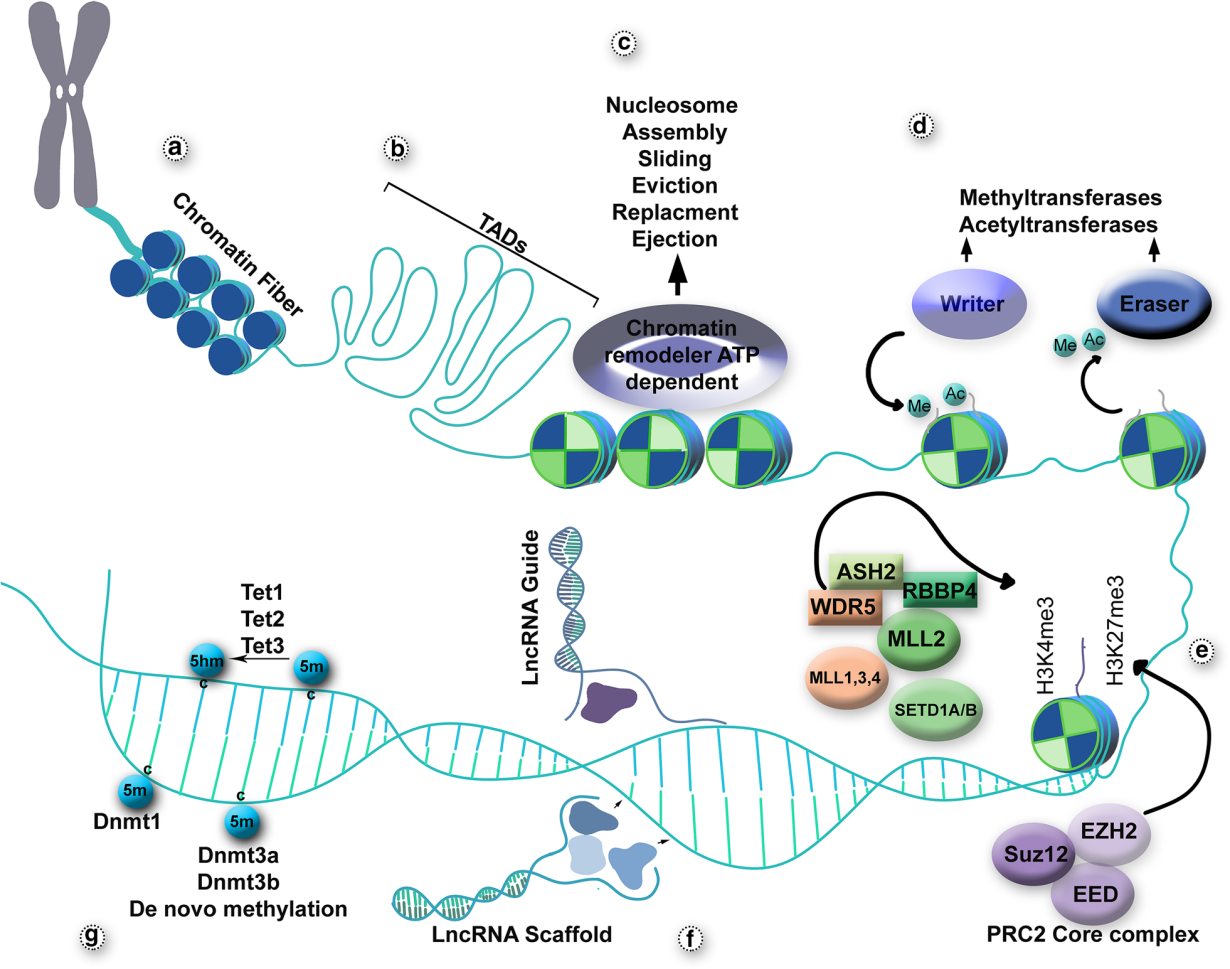
Schematic depiction of the various layers of epigenetic regulation involved in cell fate determination and differentiation events. **a** Chromatin folding can be considered as the first layer of transcriptional regulation; nucleosomes are assembled and form 30-nm fibers. **b** Topologically associating domains (TADs) are genomic regions spanning 0.2 to 1 Mb considered as defined regulatory and structural units. **c** Binding of ATP-dependent chromatin remodelers to chromatin exposes sites for transcriptional regulation. **d** Histone tails are subjected to different covalent modifications mainly on their lysine residues. Enzymes known as “writers” (acetyltransferases and methyltransferases) are responsible for adding these modifications, while “erasers” (deacetylases and demethylases) remove them. **e** Various proteins and protein complexes are involved in establishing bivalent histone marks; the most abundant subunits are components of the PRC2 core complex, which catalyze H3K27 methylation and MLLs, which catalyze H3K4 methylation. **f** lncRNAs fine-tune gene expression in several ways, such as guiding proteins to a specific location in the genome or acting as a scaffold for other regulatory components. **g** DNA methyltransferases, such as Dnmt1, Dnmt3a and Dnmt3b add or maintain methyl groups on cytosine residues (5-methylcytosine), while members of the ten-eleven translocation (Tet) family catalyze the oxidation of 5-methylcytosine to 5-hydroxymethylcytosine, which can then be further converted to other intermediates or removed to demethylate the DNA

During retinal development, distinct neuronal subtypes are generated sequentially, and in a precise order, from the same pool of retinal progenitor cells (RPCs) [1]. Progenitor traits are gradually lost during neurogenesis as RPCs acquire specific neuronal characteristics and differentiate. Deciphering the molecular events that occur during the transition from RPCs to differentiated neurons is critical for understanding how the retina is built during development. Conceptually, cell fate determination and lineage maintenance rely on a complex interplay between regulatory mechanisms at both the DNA and chromatin levels. Indeed, processes such as post-translational histone modifications, chromatin remodeling, histone turnover, DNA methylation, and the activity of long non-coding RNA (lncRNAs) affect nuclear processes like chromosome compaction, chromatin accessibility, transcription and DNA repair that in turn can modulate cell fate specification and differentiation events during development (Fig. 1) [2–4]. There is a growing understanding of this complexity in a variety of central nervous system (CNS) tissues, including the retina [5, 6].

**Fig. 2:**
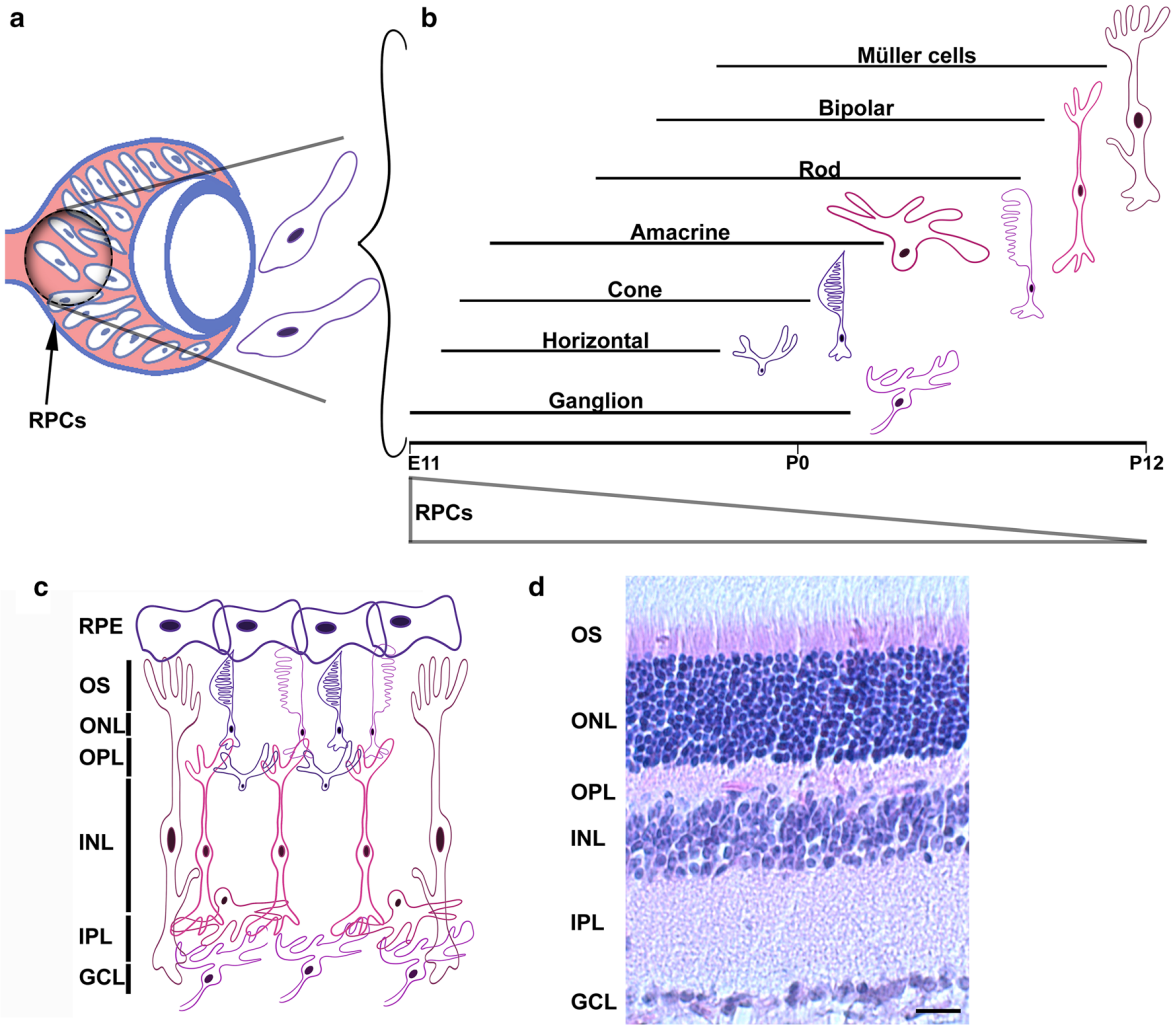
Vertebrate retinal development. **a** Cartoon of the developing retina. Early in development, the retina is composed of multipotent retinal progenitor cells (RPCs). **b** Over time, RPCs give rise to the seven cell types of the mature retina and they do so with precise spatio-temporal precision. Retinal ganglion cells are generated first, followed by horizontal cells, cones, amacrine cells, rods, bipolar cells and finally Muller glia. As development proceeds, the competency of RPCs to give rise to each of these cell types becomes further restricted. **c** Cartoon of the laminar architecture of the mature retina, wherein differentiated neurons are precisely organized into three principal cellular layers: the outer nuclear layer (ONL), inner nuclear layer (INL) and ganglion cell layer (GCL). These layers are separated by the synaptic layers: the outer plexiform layer (OPL) and inner plexiform later (IPL). The retinal pigmented epithelium (RPE) lies at the posterior of the eye, supporting the retina. **d** Histological section (H + E stained) of a 13-week-old mouse retina highlighting these retinal layers. Scale bar = 10 um

The retina is a highly accessible part of the vertebrate central nervous system. As such, it constitutes an ideal organ in which to perform experimental manipulations to better understand the epigenetic regulation of neural development. The mature retina is composed of seven cell types (Fig. 2): rod and cone photoreceptors; interneurons—horizontal cells, bipolar cells and amacrine cells; output neurons—retinal ganglion cells (RGCs); and a single glial cell type—the Muller glia. These cells are arranged into a precise laminar organization within the mature retina, generating five principal layers: three of these are cellular—the outer nuclear layer (ONL), inner nuclear layer (INL) and ganglion cell layer (GCL) and two are primarily synaptic—the outer plexiform layer (OPL) and inner plexiform layer (IPL). The retina develops from a pool of RPCs and all retinal cell types and sub-types are generated from this same population of RPCs in a relatively short time window. This rapid development from a common pool of cells is accompanied by coordinated changes in chromatin structure to accommodate developmental transitions and differentiation waves [4, 7]. In this review, we focus on the epigenetic regulation of retinal development and maintenance, taking a broad view of epigenetic processes and discuss several examples of functional crosstalk between these regulatory processes during retinal development. Epigenetic analyses during retinal development are still in their early stages and therefore many questions remain to be answered. We touch on several of these throughout the review and discuss several examples of potential epigenetic underpinnings of ocular disease.

## Histone modifications and chromatin state dynamics during retinogenesis

In eukaryotic cells, histones are proteins that function to condense the DNA into nucleosomes. The major histones are the core histones, H2A, H2B, H3 and H4, along with the linker histones H1/H5 [8]. Chromatin states are dynamic and are defined by the individual or combinatorial chemical modifications on the N-terminal tails of the histones, which lead to transcriptional activation or repression [9]. Many studies have investigated histone modifications during retinal development using histological and biochemical approaches, and more recently by employing a variety of next-generation sequencing technologies. Using immunohistochemical techniques, H3 lysine 4 trimethylation (H3K4me3), a mark of active chromatin, and H3 lysine 27 trimethylation (H3K27me3), a mark of repressed chromatin, were detected in the embryonic and adult mouse retina [10]. However, the distribution of histone modifications is highly dynamic and cell type-specific; for example, genes expressed in mature rod photoreceptors have a unique accumulation pattern of H3 lysine 4 dimethylation (H3K4me2) at the transcription start site (TSS), which is associated with increased transcription [11]. Indeed, histone marks such as H3K4me1, H3k4me2, H3k4me3, H3k27me3, H3k27ac are dynamically enriched at specific loci to regulate the temporal kinetics of gene expression. H3K4me1 and H3K27ac are enriched at enhancers of genes that are actively transcribed in RPCs, and absent from those regulating genes expressed in mature cones and rods [7]. Conversely, during early postnatal stages and in mature rods and cones, H3K27ac and H3K4me3 accumulate at active promoters controlling the expression of rod- and cone-specific photoreceptor genes [7, 12].

Adding further complexity to chromatin state logic, studies have found that the active H3k4me3 and repressive H3K27me3 modifications may coexist on genomic loci [13]. Such “bivalent” marks have generated much interest over the last decade particularly because of their roles in stem cells [13]. Bivalent domains are established in stem cells on silenced developmental genes rendering them in a poised state, that is subsequently resolved into permanent repression or activation during differentiation [14]. It seems that the bivalent domain is also utilized during retinogenesis in a similar manner to control retinal gene expression during development. For instance, during early development, the promoter of syntaxin 3 (*Stx3*), a gene expressed in mature rod cells, is enriched with bivalent modifications (i.e., H3K27me3 and H3K4me3/H3K27Ac), while in differentiated rods, H3K27me3 is depleted at the promoter [4].

To facilitate an understanding of the dynamics of histone marks in a biologically meaningful way, a machine-learning algorithm, called chromatin Hidden Markov Modeling (chromHMM) was developed to catalogue different histone marks into groups or “states” [15]. While this statistical method is far from perfect, it enables a better understanding of the dynamics of chromatin signatures during development. chromHMM analyses of retinal development and in retinal pigment epithelium (RPE) cells have revealed dynamic changes in chromatin regulation [4, 16]. A caveat to the RPE study is that it used a limited number of histone marks to generate chromatin states. Still the study revealed that the majority of promoters possess open or active chromatin (i.e., an empty or permissive state) [16]. In the developing retina, 11 chromatin states were identified in human and mouse from ChIP-Seq data of 11 histone marks and chromatin-associated proteins [4]. chromHMM modeling demonstrated that the percentage of a gene (+/−2 kb) or promoter (TSS-2 kb) containing bivalent histone marks at P0 is two times larger than P21, which suggests that the majority of the fate determination genes are in a poised state, and upon a specific trigger, the repressive mark would be erased [4]. However, these data were obtained from whole retinae rather than distinct retinal cell types and therefore it will be important in future analyses to assess chromatin states from each cell type and at different time points during their development to fully understand the regulatory logic imposed by chromatin modifications on these fate decisions and differentiation events.

## Histone variants

Histone variants are histones that replace core histones to further modulate nucleosome structure and activity. H2A.B, H2A.Z, H2A.X, macroH2A, and H3.3 are the most common variants and these are highly conserved across species [17]. While little is known about how these variants might contribute to retinal development, many histone variants are expressed in neural tissues, pointing towards a possible role in neurogenesis. For example, recent studies have shown that mutations in the histone variant H3.3 (but not canonical histone H3) drive pediatric brain tumors by promoting stem cell-like properties within glioblastoma cells [18, 19], underscoring the need to understand their contribution to the transition from proliferation to differentiation during neural differentiation. Knockdown of *H2A.Z* in embryo bodies (EBs) results in impairment of neural differentiation in the presence of retinoic acid (RA), suggesting that H2A.Z plays a critical role in cell fate transitions and lineage commitment [20]. Histone H2A.X is also involved in the DNA damage response and DNA repair during hypoxia-dependent neovascularization. Under hypoxic conditions, H2A.X and its C-terminal phosphorylated form, gamma-H2A.X, play an essential role in maintaining endothelial cell proliferation [21]. In *H2A.X*^−/−^ mice subjected to hypoxic conditions, retinal angiogenesis decreased when compared to wild-type mice [21]. This decrease in retinal neovascularization correlated with an increase in endothelial cell apoptosis and decreased endothelial cell proliferation, suggesting a possible avenue for development of therapeutics aimed at preventing neovascularization in diseases like retinopathy of prematurity and diabetic retinopathy [21].

In addition to the core histones and their variants, data also point toward a possible role of the linker histone (H1) and its variants during retinal development. Studies on rod photoreceptor development in mice have shown that the total level of histone H1c increases, concomitant with the increase in the length of the nucleosomal repeat from 190 to 206 bp [22]. Triple *H1c/H1e/H10* knockouts result in decreased heterochromatin area, larger nuclei and impairment of the structural integrity of rods [22], possibly linking defects in chromatin condensation to rod physiology. Among all histone H1 variants, Hist1h1c has the highest expression levels in the mouse retina [23]. Knockdown of *Hist1h1c* in Ins2^+/+^ and Ins2^+/−^ mice (a diabetic retinopathy model) resulted in increased expression of autophagy markers, such as LC3B-I and LC3B-II, and repressed the diabetes-induced transcription of proinflammatory factors [23]. On the other hand, overexpression of *Hist1h1c* in the retina resulted in ectopic induction of autophagy and diabetic retinopathy-like phenotypes. In this model, the number of cells in the GCL decreased and the INL and IPL thickness were reduced, suggesting that Hist1h1c levels may contribute to the regulation of autophagy, inflammation, and neuron loss, similar to diabetic retinopathy [23]. Considering the importance of histone variant deposition, turnover, and histone chaperones as regulators of chromatin dynamics, elucidating their role in retinal development is likely to reveal important roles during retinal cell fate determination and lineage commitment.

## Histone-modifying enzymes and their functions during retinal development

Several enzymes orchestrate post-translational modification (PTM) of histones through the addition or removal of different chemical groups on histone tails, and these are critically important in regulating chromatin structure and function. Most histone-modifying enzymes are ubiquitously expressed, yet it has become clear that their roles are context-specific [24]. Furthermore, these proteins can have chromatin-independent functions in a variety of cellular process, further underscoring the importance of studying their tissue-specific functions [25, 26]. Here, we discuss some of the known roles of histone modifications and chromatin-modifying enzymes during retinal development.

### Modifying enzymes: writers

Histone acetyltransferases and methyltransferases are two main classes of histone “writers” that act as molecular switches to maintain the active or silenced states of target loci, respectively [27]. Histone acetyltransferases add acetyl (CH_3_CO) groups, while methyltransferases catalyze the addition of a methyl (CH_3_) group to lysine residues on histone tails [28]. Of those, the evolutionarily conserved Polycomb (PcG) and Trithorax (TrxG) were first discovered in *Drosophila melanogaster* as master regulators of Hox gene expression and found to play important roles in embryonic stem (ES) cell self-renewal, cell fate choice, proliferation, apoptosis, cell cycle regulation, plasticity and regeneration [29–31]. PcG forms at least two main repressive complexes: Polycomb repressive complex 1 (PRC1) and Polycomb repressive complex 2 (PRC2) [32, 33]. Methylation of lysine 27 on histone H3 (H3K27me) is catalyzed by the activity of PRC2; as a consequence, PRC1 is recruited, and this leads to a cascade of gene-silencing events [34, 35].

PRC1 and PRC2 complex functions have been studied during retinal development. For instance, the PRC1 component Bmi-1 is expressed in immature RPCs and differentiated cones, where it is required for cone maintenance [36]. Indeed, *Bmi-1* deficiency resulted in misregulated proliferation and self-renewal of RPCs [37]. Rybp is an evolutionarily conserved zinc finger protein that is a non-canonical PRC1 member and functions as a transcriptional repressor, acting through histone modification. Rybp deficiency is embryonic lethal during early stages of development [38, 39]; however, studies of chimeric mice consisting of Rybp mutant and wild-type cells indicate that Rybp function is essential for anterior segment and retinal development [40]. Loss of a single *Rybp* allele leads to retinal coloboma (a failure of optic fissure closure), while overexpression of *Rybp* in the lens causes abnormal fiber cell differentiation, and lens opacification [40]. Moreover, ubiquitous Rybp overexpression in the eye results in abnormal retinal folds and corneal neovascularization [40]. Samd7, another PRC1 component, is predominantly expressed in the photoreceptor layer of the developing retina and loss of its function leads to derepression of non-rod genes, likely due to loss of the repressive mark H3K27me3 [41]. Furthermore, Samd7 may serve as transcriptional repressor involved in fine-tuning of the transcription factor cone–rod homeobox (Crx), a critical transcriptional regulator of photoreceptor development [42].

The roles of the PRC2 and its core subunits have also been investigated during retinogenesis [43–45]. Enhancer of zeste homolog 2 (Ezh2) serves as a catalytic subunit of PRC2 and functions primarily in the establishment of lysine H3K27 modifications that repress expression of target loci [46]. Ezh2 is strongly expressed in the embryonic retina and expression decreases after birth [47]. Ezh2 directly or indirectly influences retinal cell differentiation and maturation as conditional knockout of *Ezh2* in retinal progenitor cells results in microphthalmia, reduction in postnatal progenitor proliferation, and accelerated differentiation of rods and Muller glia [43, 45]. Beyond changes in histone modifications at target loci, the expression of several critical rod genes such as *Pnr, Nrl*, and rhodopsin is also changed during embryonic stages following *Ezh2* knockout [45]. Interestingly, the deletion of *Ezh2* in retinal progenitors at embryonic stages also results in gradual photoreceptor degeneration throughout postnatal life, possibly through the de-repression of *Six1* and photoreceptor-related genes [48].

The core component of PRC2, Eed (Embryonic ectoderm development), also plays a crucial role during retinal development. Conditional knockout of *Eed* at postnatal stages results in severe defects in retinal lamination, a depletion of RPCs and an increase in the proportion of early-born amacrine cells at the expense of late-born cell types such as Müller glia [43]. In conditional knockouts of Eed, H3K27me3 and H3K4me3 are enriched on the promoters and gene bodies of amacrine-specific genes, and this activity is essential for amacrine cell fate determination and differentiation [43]. Similar to Ezh2 knockout, *Eed* conditional knockouts also possess reduced RPC proliferation and increased cell death at postnatal stages, which resulted in disruption of late-born retina cells such as Müller glia, bipolar, and rod photoreceptor generation [43]. These results are consistent with those in *Drosophila* where mutation in *E(z*) and *Su(z)12*, the *Drosophila* counterparts of the vertebrate PcG components Ezh1/Ezh2 and Suz12, respectively, result in eye discs that are substantially smaller than the wild-type [49]. In *Drosophila*, PcG targets the majority of master regulator transcription factor genes involved in eye development (e.g., *eya, so, dac, eyg*) and similarly, in humans and mice PRC1 and PRC2 also target retinal specification and patterning genes (e.g., *SHH, WNT3A, SIX1, PAX3, PAX6, Six6, Pax6, Shh, Otx1*) [50]. These data indicate that PRC2 acts on broad ranges of retinal and non-retinal genes and has stage-specific functions during retinal differentiation and maturation.

Another repressive enzyme is G9a, a nuclear histone lysine methyltransferase (HMT) that mainly catalyzes histone H3 lysine 9 mono- and di-methylation, which are linked to transcriptional silencing [51]. G9a and PRC2 have several common target genes that encode proteins which are mostly involved in neural development and differentiation [51]. Conditional *G9a* knockout results in retinal defects including the formation of photoreceptor rosettes, mislocalization of RPCs, prolonged RPC proliferation and elevated cell death [52]. Whether G9a and PRC2 activities are coordinated during retinal development remains to be explored.

TrxG proteins mediate histone methylation and chromatin remodeling activity, which leads to the maintenance of chromatin in an active mode; moreover, TrxG family proteins mainly act via nucleosome remodeling or histone modification, the latter of which includes H3K27ac, H3K4me3 and dimethylation of Lys36 on histone H3 (H3K36me2) [53]. Mixed-lineage leukemia 1 (Mll1), a TrxG member, acts as an H3K4me1-3 writer [54, 55]. Conditional knockout of *Mll1* in mouse results in deficits in visual function, morphological defects in the retina that include altered cell type composition, a reduction in INL thickness, likely due to reduction of the progenitor cell pool, and defects in horizontal cell morphology and survival [56]. Mll3/4 is the mammalian counterpart of *Drosophila* trithorax-related (Trr), and functions as an H3K4 monomethyltransferase [57]. Trr activity can suppress cell growth in *Drosophila* eye imaginal discs by reducing H3K4 monomethylation and thereby affect multiple growth-promoting pathways [57, 58]. Given that Mll complexes are associated with transcriptional activation, it is likely that their function is mediated by transcription factors that promote cell type-specific differentiation programs. Indeed in the developing photoreceptors of the mouse retina, NRL (a key transcription factor that promotes rod photoreceptor differentiation) recruits the histone acetyltransferase, Kat5 (Tip60), to activate transcription of the rod-specific downstream targets *Ppp2r5c* and *Rhodopsin* via H3/H4 acetylation [59], while the cone–rod homeobox transcription factor Crx, facilitates the binding of the histone acetyltransferase p300/CBP to Crx-regulated photoreceptor promoters [60]. Conditional knockout of *p300/CBP* resulted in misregulation of photoreceptor-related genes via reduction of histone H3/H4 acetylation [60]. Beyond the developing retina, p300/CBP is also crucial for lens induction, as conditional knockout of *p300/CBP* in the developing lens placode resulted in aphakia (absence of the lens) [61]. This phenotype may result from reduced expression of *Six3, Sox2, Otx1,* and *Pitx3*, which together play pivotal roles in regulating lens formation [61].

Collectively, the studies discussed above demonstrate the importance of histone writers in modulating several aspects of retinal development. While most of them seem to be essential for retinal proliferation, their roles during retinal differentiation and cell type specification vary, which highlights the need for in depth analysis of their genome-wide roles in regulating distinct retinal cell fate determination events during development.

### Modifying enzymes: erasers and readers

The main types of histone “erasers” are histone demethylases and histone deacetylases. These enzymes remove acetyl or methyl groups from histone lysine residues [62–66]. The Jumonji family of histone demethylases and lysine-specific histone demethylase 1 **(**LSD1) proteins act as erasers of lysine methyl marks on histones [62, 64]. Histone deacetylases (HDACs) are responsible for removing the acetyl group from lysine residues on histone proteins [66]. With regard to “readers”, numerous proteins containing domains such as bromo-adjacent homology (BAH), chromodomain, PHD (plant homeodomain), tandem Tudor domain (TTD), double chromodomain (DCD), WD40 and the zinc finger CW (zf-CW) have been recognized as readers of methyl lysine [67]. Bromodomain proteins are the most well-characterized acetyl lysine reader [67].

Members of the Jumonji protein family possess a conserved JmjC domain which functions in histone demethylation via a mechanism involving an oxidative reaction that requires α-ketoglutarate (αKG) and iron Fe(II) as cofactors [68]. Twenty-seven different JmjC domain proteins have been detected in the human genome the functions of most of which have yet to be explored, including in the retina [68]. Kdm6b, which demethylates H3K27me3/me2, is expressed in the INL during late stages of murine retinal development [69]. Knockdown of *kdm6b* results in a decrease of *Bhlhb4* and *Vsx1* expression, critical transcription factors in the differentiation of rod-ON bipolar cells and cone-OFF bipolar cells. The loss of the Kdm6b in mouse retina leads to failure in the differentiation of these two subsets of bipolar cells [69, 70], while pharmacological disruption of Kdm6b causes increased RPC proliferation and reduction of bipolar cells [71]. In the developing *Xenopus* eye, Kdm6b is expressed in the inner regions of optic cup, but its role in the optic cup has not been functionally investigated [72].

In *Xenopus*, the lysine-specific histone demethylase 5C (Kdm5c) is also expressed in the embryonic eye and knockdown of Kdm5c results in smaller and deformed eyes, perturbed retinal lamination and abnormal RPE formation [73]. Knockout of *Jmjd6* (*Ptdsr*) in mouse results in severe disruption in eye formation, with defects ranging from impaired retinal neuron differentiation to complete unilateral or bilateral anophthalmia [74]. Fbxl10 (Kdm2b) is a JmjC domain-containing histone demethylase; homozygous mutation in *FbxI10* results in neural tube closure defects, an expanded retina and retinal coloboma [75].

LSD1 (Kdm1a) was the first protein lysine demethylases discovered [76]. In the mouse retina, Lsd1 is highly expressed at postnatal stages and expression gradually decreases over time [77]. Pharmacological inhibition of Lsd1 in retinal explants at early postnatal stages results in misregulation of genes associated with progenitor function and impaired rod formation [78]. With respect to retinal disease, global levels of H3K27me3 are increased in *rd1* mice, a model of retinitis pigmentosa, and application of the histone methyltransferase inhibitor, 3-deazaneplanocin A (DZNep) to postnatal retinal explants results in a reduction of calpain activity (a marker of dying cells), delayed photoreceptor loss and improved the light-adapted electroretinogram. These data suggest that modulation of histone modifications could be a potential therapeutic candidate for treating retinal degenerative diseases [79].

Histone deacetylases also play a role in retinal development. It has been shown pharmacological inhibition of Hdac1 activity in mouse impairs rod photoreceptor differentiation [80]. Mechanistically, it is thought that these defects result, in part, through the misregulation of progenitor-specific genes such as *Hes1* and *Vsx2* via concomitant increases in acetylation of histone 4 lysine 12 (H4K12) and H3K9 [80]. In zebrafish, *hdac1* mutation resulted in reduction in *cyclin D* and* E* expression and failure of RPCs to exit the cell cycle [81]. *hdac1* function is vital for retina and optic stalk differentiation; in *hdac1* mutants, retinal lamination is disrupted and differentiated RGCs and photoreceptors are absent [81]. Sirt1 is a nicotinamide adenosine dinucleotide (NAD)-dependent deacetylase; knockout of *Sirt1* in mouse resulted in abnormal closure of the optic fissure, thinner and disorganized plexiform layers, and defects in the formation of photoreceptor inner and outer segments. These defects were thought to potentially result from hyperacetylation of p53 [82]. In summary, while the majority of lysine demethylases and deacetylases have not yet been well studied in the retina, these findings suggest that they play a critical role during ocular development and their misregulated activity could potentially contribute to retinal disease.

## Nucleosome remodeling and chromatin accessibility

### Chromatin remodeling and structure

Chromatin remodeling complexes play pivotal roles in neural development and differentiation [83]. During neural differentiation, chromatin remodeling factors change their subunit composition concomitant with transition from proliferation to differentiation, underscoring the highly specific function of these subcomplexes during neural differentiation [84]. Indeed, several studies have identified distinct roles for chromatin remodeling enzymes in the retina. BRG1 (Smarca4), a subunit of the SWI/SNF complex, is essential for retinal lamination and overall retinal development in mice and zebrafish [85–87]. Inactivation of *Brg1* in mice results in microphthalmia and defects in photoreceptor differentiation [87]. Snf2h (Smarca5), another subunit of SWI/SNF, is critical for lens differentiation via maintaining the balance between epithelial and fiber cell differentiation in mouse; however, roles in retinal development have not yet been identified [88]. Brm, a SWI/SNF subunit, is essential for RGC differentiation and acts by modulating *Brn3b* expression [89]. During embryonic stages, Baf60c, another subunit of SWI/SNF, is expressed in retinal progenitors; overexpression of Baf60c in RPCs results in enhanced proliferation [90]. Chromodomain-helicase-DNA-binding protein 7 (CHD7) plays a role in chromatin organization and is mutated in a number of human diseases including CHARGE (coloboma of the eye, heart defects, atresia of the choanae, retardation of growth/development, genital abnormalities, and ear anomalies) syndrome. In zebrafish *chd7* morphants, eyes are smaller and retinal organization is disrupted with a reduction in RGC numbers and a lack of photoreceptor layer maturation [91]. Conditional *Chd7* knockout in mice also resulted in dysmorphic eyes with unrecognizable optic cups and small lenses [92]. There are numerous other chromatin remodeling proteins and it will be of interest to determine whether they play roles during retinal development and/or in specific retinal cell types, as well as during the progression of retinal diseases.

### High-order chromatin structure and gene regulation

Chromatin compartmentalization and nuclear localization also play an essential role in retinal development. The nucleus of retinal neurons, just like any other conventional nucleus, has centralized euchromatin, and condensed heterochromatin adjacent to the nuclear lamina [93]. Rod cells, however, have an inverted nuclear organization where heterochromatin is located centrally. Evidence indicates that this unique chromatin distribution contributes to a reduction in light loss, thus improving the ability of nocturnal animals to see in the dark [94, 95]. The molecular mechanisms contributing to nuclear organization have begun to be revealed, as nuclear envelope proteins, such as lamin B receptor (Lbr) and laminA (Lmna), are responsible for establishing the conventional nuclear organization [94]. During photoreceptor differentiation in mice, rod precursors gradually lose the expression of *Lbr* and *Lmna* leading to inverted chromatin architecture in fully differentiated rods [94]. Indeed, in *Lbr*^*ic−J/ic−J*^ mutant mice, heterochromatin is not tethered to the nuclear lamina in inner nuclear layer cells, while overexpression of Lbr in the retina disrupts the inverted architecture of the rods [93]. The relevance of this unique nuclear architecture on gene expression and retinal function remains poorly understood; indeed, conditional knockout of Lbr in mice reveals only minor differences in gene expression between knockouts and littermate controls and no effect on visual function [93]. Finally, chromatin-modifying enzymes may also contribute to the regulation of nuclear architecture; for example, Casz1 is a transcriptional factor that interacts with the Polycomb members Ring1b, Suz12, and Hdac2, and depletion in the mouse retina results in reduced expression of lamin A/C and chromatin inversion in rods [96].

The transition of euchromatin to heterochromatin has a direct influence on the regulation of gene expression in the retina; for example, *Car10*, a gene that is expressed in bipolar cells but not in rods, is sequestered to facultative heterochromatin in rod nuclei [93]. While this type of unique localization is not a common feature of all genes that are repressed in rod cells it might provide an added level of gene regulation to repressed genes by limiting transcription factor accessibility to those genes [93]. Fluorescent in situ hybridization (FISH) is an invaluable method to visualize genome organization that has been leveraged to shed light on the dynamics of promoter–enhancer interactions during retinal development and how they are influenced by chromatin compartmentalization. For example, it has been shown that a loss of interaction between *Sox2* and its enhancer during retinal development is associated with sequestration of the Sox2 enhancer into facultative heterochromatin [93]. Through techniques like this, predicting gene expression, non-coding DNA regions and *cis*-regulatory elements based on genome localization and accessibility is a powerful approach to study the genomic landscape.

Analysis of open chromatin with methodologies such as DNase hyper-sensitivity and ATAC-seq coupled with assays to investigate 3D chromatin structure (i.e., Hi-C) have provided insights into the relationship between chromatin structure and gene expression during retinal development and in distinct retinal cell types. Indeed, the genome-wide mapping of accessible chromatin with ATAC-seq in rods and cones of mature mice demonstrated that in rods, thousands of loci are selectively closed relative to those in blue and green cones. This may be due to the regulatory role of *Nrl*, as in *Nrl*^−/−^ photoreceptors, the global chromatin accessibility of rods is similar to other cell types [97]. The LIM homeodomain transcription factor *Lhx2* is expressed in the developing mouse retina and in specific cell types of the adult retina [98]. In RPCs, Lhx2 is involved in regulation of local and global chromatin accessibility; conditional *Lhx2* knockout in RPCs results in loss of accessibility of Lhx2 targets [98]. It was proposed that Lhx2 regulates chromatin accessibility by competition for nucleosome occupancy, which could have a substantial impact on the regulation of gene expression [98]. The 3D chromatin landscape of the mouse retina has been mapped during embryonic (E14.5), postnatal (P0) and adult stages by ultra-deep in situ Hi-C and the results of this comprehensive analysis demonstrated that the enhancer-promoter interactions and chromatin compartments are dynamic at different developmental stages [93]. Integrating single-cell ATAC-seq (scATAC-seq) and single-cell RNA-seq with these data identified putative developmental stage-specific super-enhancers (SE), including one regulating *Vsx2* expression, deletion of which resulted in a loss of bipolar cells [93].

Analysis of chromatin accessibility and gene expression have also been recently performed in developing human retinae and human (h) iPSC-derived retinal organoids, expanding our understanding of human retinal development as well as the potential uses and limitations of iPSC-derived retinal organoids and their constituent cell types in modeling and treating human retinal disease. For example, integration of RNA-Seq data from the developing human retina with DNase-seq data at two fetal stages (Day 74 and Day 125) revealed a strong association between chromatin accessibility and gene expression, which could then be used to predict putative gene regulatory networks regulating human retinal development [99]. A comparative study of chromatin accessibility (via ATAC-Seq) and gene expression between developing human retinae from gestational week (GW) 6 to 25 and similarly staged hiPSC-derived retinal organoids revealed that while chromatin accessibility and gene expression in retinal organoids are quite similar overall to the in vivo retina, there are, however, stage-specific differences [100]. In particular, accessible chromatin regions during the middle stages of human retinal development (GW10 to 25) were accessible only in late stage (week 23 to 30) hiPSC-derived retinal organoids, and these regions were enriched in genes regulated neurogenesis and neuronal differentiation [100]. A similar phenomenon is observed in mice when ATAC-Seq data are compared from native retinae and iPSC-derived retinal tissue; here, modest stage-specific differences in chromatin accessibility between the two systems were also identified [101]. When integrating human ATAC-Seq data and ChromHMM data from human and mouse [4], bivalent H3K4me3 and K3K27me3 marks were noted to be enriched in human neurogenesis genes that were accessible during the middle stages of development, but not in those in mouse at comparable stages, suggesting differences in the epigenetic regulation of retinal development between mice and humans. Finally, in considering the reprogramming efficiency of mouse retinal neurons into iPSCs and retinal organoids, epigenetic memory appears to play a key role where retinal neurons with lower efficiency of reprogramming, possibly as a result of sustained epigenetic memory, were able to produce more differentiated retinae than those with higher reprogramming efficiency [102], indicating that epigenetic memory may be an important consideration in selecting and generating cells for potential use in therapeutic applications.

From a disease perspective, *Abca4*^−/−^*Rdh8*^−/−^ double knockout (dKO) mice are a photosensitive model of stress-induced photoreceptor degeneration, which is widely used for modeling age-related macular degeneration (AMD) pathology [103]. Following prolonged exposure to bright light, the retina and RPE/choroid of *Abca4*^−/−^*Rdh8*^−/−^ dKOs manifest a global decrease in chromatin accessibility over time, which results in gene expression changes that drive apoptosis. Importantly, while the global chromatin accessibility decreased in this model after bright light, the accessibility of genomic regions surrounding key inflammatory response genes increased following photobleaching, suggesting that changes in chromatin accessibility could contribute to reactive gliosis and apoptosis in the retina [103]. Genomic analyses of chromatin accessibility of retinal and RPE cells from AMD patients revealed a broad decrease in accessibility with AMD progression, but interestingly, accessibility decreased in RPE cells at an earlier stage than retinal cells, suggesting that defects in RPE cells is critical in disease onset [104].

### DNA methylation

DNA methylation contributes to dynamic regulation of chromatin architecture and the regulation of gene expression in many developing organs and tissues. DNA can be covalently modified through addition or removal of a methyl group predominantly to the cytosine base found in the dinucleotide sequence 5′CpG3′. DNA methylation is a heritable epigenetic mark established and maintained through the activity of DNA methyltransferase (DNMT) enzymes. Methylation is reversible where the methyl group can be removed through several mechanisms, including via the activity of ten-eleven translocase (Tet) proteins [105]. DNA methylation plays a role in developmental processes such as genomic imprinting, X-chromosome inactivation, cellular aging, cell differentiation, cell death, and gene silencing [106, 107].

In the retina, mature retinal neurons possess distinct cell-specific DNA methylation patterns [108]. For example, in the mouse retina, rod and cone cells have different methylation landscapes wherein rods possess hypomethylated DNA and a closed chromatin state relative to cones [7]. The associated genomic regions appear to be active in early developmental stages but diminish their expression in mature rods [7]. In mice, photoreceptor fate determination is tightly regulated by DNA methylation. In RPCs, the promoter and genomic regions controlling the expression of rod and cone-specific genes are highly methylated; however in mature photoreceptors these regions are unmethylated or hypomethylated relative to RPCs [109]. Recently, it has also been documented that global methylation patterns may change with age in rods; age-related DNA methylation changes are not random, but rather, they localize to specific regions, such as rod regulatory elements, which may be responsible for controlling transcription of genes involved in rod homeostasis [110].

Functional analyses of DNA methylation enzymes have implicated DNA methylation in a variety of processes regulating retinal development. Morpholino knockdown of *dnmt1* and *dnmt3* (*dnmt3bb.2*) expression in the developing zebrafish retina revealed that both genes are essential for neuronal differentiation and retinal lamination [111, 112]. Loss of function mutations affecting *dnmt1* in zebrafish also resulted in progressive degeneration of the ciliary marginal zone (CMZ), an endogenous stem cell containing zone of the peripheral retina [113], as well as severe defects in lens and retina formation [114]. Morpholino knockdown of *dnmt2* (*trdmt1*) in zebrafish, resulted in mild microphthalmia, retinal lamination defects, RPE malformation, and reduced expression of retinal cell type markers like *atoh7*, *neurod, and zash1a*, suggesting that DNA methylation may be involved in the differentiation of retinal neurons [115]. Indeed, conditional knockout of *Dnmt1* in the developing mouse retina hampered the differentiation and maturation of retinal neurons and led to rapid photoreceptor degeneration [116]. Furthermore, *Dnmt1* conditional knockout in the RPE resulted in the absence of cone outer segments [117]. Mouse triple knockouts for *Dnmt1, Dnmt3A, Dnmt3B* resulted in severe retinal phenotypes such as the lack of photoreceptor outer segments, reduced outer plexiform layer size, reorganization of synaptic layers, and overall defects in visual function [118].

As stated above, DNA methylation is transient and demethylation can be initiated through the activity of the Tet enzymes which convert the methyl group to hydroxymethylcytosine and subsequently to formylcytosine and carboxycytosine, which is then removed through base excision repair [105]. These intermediates may also play distinct roles during cell specification and differentiation events during development [119]. Tet activity has been recently studied in the zebrafish retina; loss of function mutations in *tet2* and *tet3* identified a requirement for Tet activity during retinal neuron formation, possibly by modulating Wnt and Notch pathway activity [120]. Morpholino knockdown of *tet3* in *Xenopus* resulted in anophthalmia; *tet3* depletion leads to misregulation of *pax6, rx and six*3 expression, suggesting a potential role during eye induction [121].

Changes in DNA methylation patterns at loci regulating eye formation may be important evolutionarily. Regression of eyes is a morphological feature of cave-adapted animals such as cavefish; loss of the eye may result as an adaptation to conserve energy in a nutrient-deficient environment [122]. The *Astyanax mexicanus* cave morph evolved from a surface morph and has lost its eyes [123]. In the eyeless morph, eye tissue degeneration is witnessed by 5 days of development, and in adulthood, the eye is absent. It was recently shown that *dnmt3bb.1* is overexpressed at 54 h post-fertilization in the eye, which results in global repression of key eye development genes, such as *opn1lw1, gnb3a*, and *crx*. A single intravitreal injection of 5-azacytidine, a DNA methyltransferase inhibitor, resulted in larger eyes, well-defined lens and the formation of retinal layers in the eyeless cave morph, suggesting that DNA methylation may play a key role in eye regression in cavefish [123].

Finally, several clinical studies highlight the potential importance of DNA methylation in ocular pathologies (Table 1). Considering the importance of DNA methylation as regulator of gene expression during cell fate determination and overall retinal development and homeostasis, it will be interesting to investigate whether there are additional cell-specific or temporal-specific requirements for DNA methylation, demethylation and hydroxymethylation in the retina. Moreover, association between DNA methylation and other epigenetic processes such as the presence and/or turnover of histone variants and histone modifications also need to be evaluated during retinal development and disease to gain a fuller picture of how crosstalk between these epigenetic changes could modulate retinal development and the progression of ocular disease.

**Table 1:** DNA methylation in ocular disease

Disease	Gene	DNA modification	References
Age-related macular degeneration (AMD)	*SKI, GTF2H4, TNXB* *GSTM1/5* *IL17RC*	Hypermethylation Hypomethylation	[161–164]
Cataract	*Klotho, Cryaa, GSTP1, TXNRD2*	Hypermethylation	[165–168]
Glaucoma *Open-angle glaucoma	*Alu, Hsp70* *HERV-K	Hypomethylation Hypermethylation	[169, 170]

## Long non-coding RNA in the developing retina

RNA-based regulatory mechanisms play an important role in numerous developmental processes in eukaryotes. Besides small non-coding RNAs (ncRNAs), mammalian genomes encode numerous long non-coding RNAs (lncRNAs), which are > 200 nucleotides in size [124, 125], lncRNAs are mainly located in the nucleus, but can be distributed in the cytoplasm [126]. These molecules are defined as functional ribonucleic acids that do not encode proteins. lncRNAs participate in a number of different cellular processes that include modulation of cell cycle progression; the establishment of chromatin states, via their ability to bind and modulate the activity of chromatin-modifying proteins; modulating chromatin structure; regulating gene expression during development and differentiation; serving as transcriptional co-regulators; and roles in stem cell maintenance and cell lineage commitment [127–132].

Not surprisingly, lncRNAs are expressed in various ocular tissues and in the retina, they are expressed in specific cell types during development [133–136]. For example, in mouse retinal ganglion cells, lncRNAs such as linc-3a, linc-3b and linc-3b are associated with modulating the expression of key regulators of RGC differentiation and specification like *Math5, Isl1 and Pou4f2* [137]. Many lncRNAs exert influence on retinal cell fate specification events during development; for example, lncRNAs such as Miat, Six3os1, Tug1, Vax2os, Rncr4 and Malat-1 function in the mouse retina and Megamind and Malat-1 function in the zebrafish retina. Other lncRNAs functioning during retinal development are summarized in Table 2. Interestingly aberrant expression of lncRNAs has been associated with visual impairments such as diabetic retinopathy (Malat1, Miat, Tdrg1, Hottip, circHIPK3) [138–141], retinoblastoma (Bancr, Meg3) and glaucoma (CDKN2B-As1) [124, 141]. Mechanistically, data generated thus far suggest that most of these lncRNAs guide chromatin modifiers, mostly PRC2 and H3K9 methyltransferases, to specific genomic loci to modulate gene expression [127, 142–144]. For instance, lncRNAs, ANRIL, HOTAIR, and HOTTIP interact with, PRC1, PRC2, and MLL, respectively, which facilitate their recruitment to chromatin [145–147]. Future studies in the developing retina and in patients with visual system disorders will undoubtedly shed additional light on the cross-talk between lncRNAs and other epigenetic modifiers as well as their specific functions in the retina.

**Table 2:** lncRNAs functioning during retinal development

LncRNA	Method	Function	References
Tug1	Knockdown in neonatal mouse	Malformation or absences of outer segment photoreceptors	[171]
Vax2os	Overexpression in neonatal mouse	Disturb the cell cycle progression in photoreceptor progenitor cells	[128]
Rncr4	Knockdown in mouse embryos and postnatal mice	Control the uniformity of retina layers	[172]
Six3osi	Knockdown and overexpression in neonatal mouse	Upregulation inhibition normal photoreceptors cell specification, knockdown prevented the differentiation of bipolar cell and Müller glial	[173]
RncR2	Knockdown and overexpression in neonatal mouse	Required for amacrine cell, and Müller glia differentiation	[132]
Malat-1	Knockdown postnatal mouse	Might regulate development of retinal neurodegeneration through CREB signaling, retinal microvascular dysfunction	[174, 175]
ZNF503-AS1	Knockdown in hiPSC-RPE	Inhibit RPE differentiation	[135]
Malat-1	Morpholino knockdown zebrafish embryo	Smaller eyes, defective otic capsule	[176]
Megamind	Morpholino knockdown zebrafish embryo	Regulation of eye development	[177]

## Potential crosstalk between epigenetic mechanisms and the mitochondria

Mitochondrial function is co-regulated by the nucleus; however, mitochondria can also modulate nuclear gene expression, mainly via mitochondrial plasticity and the formation of metabolites that act as cofactors or substrates for DNA and histone-modifying enzymes [148]. For example, acetyl-CoA supplies the acetyl group for histone acetyltransferases (HATs), while S-adenosyl methionine (SAM) donates the methyl group for histone lysine methyltransferases (HMTs). Moreover, DNA methyltransferases use SAM as a methyl donor [148]. α-Ketoglutarate (α-KG) is a key intermediate metabolite of the TCA cycle and acts as a cofactor for Jumonji domain demethylases [148–150]. In addition, α-ketoglutarate is required for TET enzyme activity [148, 150–152], while the mitochondria metabolites succinate and fumarate inhibit both TETs and JMJDs [148]. Taken together, these data suggest that mitochondrial activity could influence the epigenetic landscape, but how this plays out during retinal development remains completely unknown. Given that several retinal pathologies are also associated with mitochondrial dysfunction, it is possible that their function in disease state is directly or indirectly linked to the modulation of epigenetic pathways, an area for future investigation [153–158].

## Conclusions

In this review, we have discussed the current understanding of epigenetic regulation during retinal development and focused on histones, histone modifications and variants, DNA methylation and lncRNAs, as well as the potential for crosstalk between epigenetic regulation and mitochondrial function. Importantly, while epigenetic regulation clearly plays a critical role during retinal development and in ocular disease, there is far more that needs to be learned. Indeed, for some of these epigenetic regulatory mechanisms, there is little known about if and how they operate during retinal development. Moreover, considering the cross-talk between histone variants, PcG and bivalent modifications in other cell types, it is almost certain that decoding the role of histone variants and deposition mechanisms will shed new light on cell fate determination in the retina. Furthermore, the interplay between these epigenetic mechanisms is essential for cell plasticity and likely contributes to regenerative mechanisms found in the retinae of many non-mammalian vertebrates (reviewed in [6, 159, 160]). Understanding how these epigenetic mechanisms facilitate regenerative responses and whether they can be stimulated in non-regenerative mammals should be fruitful in the quest to develop novel therapeutics to treat retinal disorders.

## Data Availability

Not applicable.
